# Supplementary material for: Oxydifficidin, a potent Neisseria gonorrhoeae antibiotic due to DedA assisted uptake and ribosomal protein RplL sensitivity
Source: bioRxiv. 2024 May 27:2024.05.27.596031. Preprint. [Version 1] doi: 10.1101/2024.05.27.596031 (PMC11160649; doi:10.1101/2024.05.27.596031)
Supplement: Supplement 1 [file NIHPP2024.05.27.596031v1-supplement-1.pdf]

bioRxiv preprint doi: https://doi.org/10.1101/2024.05.27.596031; this version posted May 28, 2024. The copyright holder for this preprint (which was not certified by peer review) is the author/funder, who has granted bioRxiv a license to display the preprint in perpetuity. It is made available under aCC-BY 4.0 International license.

a

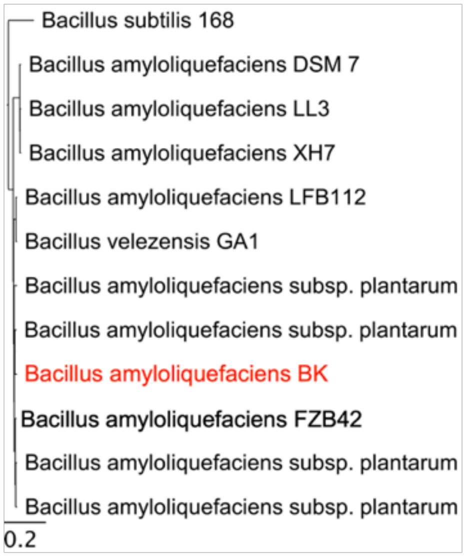

c

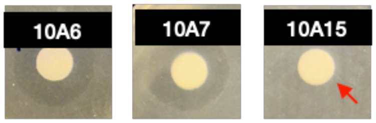

Strain	Gene Descriptor	Halted production
10A6	Wild type	None
10A7	Δ*bmyA*::EmR	Bacillomycin (mycosubtilin)
10A15	Δ*pks3KS1*::ermAM	Difficidin/Oxydifficidin

b

Transposon insertion site	Protein encoded
1	Phage SPbeta protein
2	Asp tRNA
3	Mycosubtilin synthase B
4	Transpeptidase penicillin -binding protein spoVD

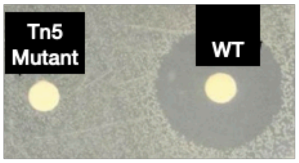

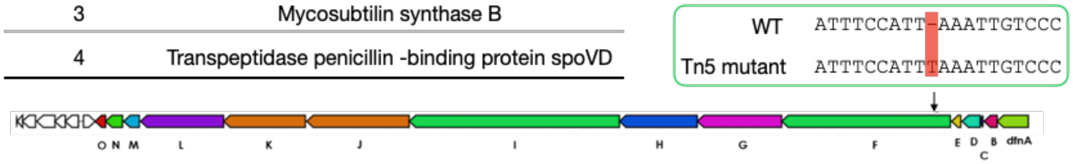

Fig. S1. Mutagenesis results of oxydifficidin-producing Bacillus spp. a. Genome-based phylogenetic tree containing *Bacillus amyloliquefaciens* BK and closely related Bacillus spp.. The tree was built using Genome Clustering of MicroScope. **b.** Disc diffusion assay of a methanol extract from cultures of WT *Bacillus amyloliquefaciens* BK (WT) and a Tn5 mutant. The test lawn was *N. gonorrhoeae*. The table shows all transposon insertion sites in the Tn5 mutant strain. The Tn5 also contains a frame-shift mutation in the *difF* gene. The red box highlights the location of frame-shift mutation in the oxydifficidin BGC. **c.** Disc diffusion assay of a methanol extract from cultures of WT and BGC knockout strains of *Bacillus amyloliquefaciens* FZB42. The test lawn was *N. gonorrhoeae*. Strain genotypes are shown in the table. Red arrow indicates that only strain 10A15 no longer produce the anti-*N. gonorrhoeae* compound.

bioRxiv preprint doi: https://doi.org/10.1101/2024.05.27.596031; this version posted May 28, 2024. The copyright holder for this preprint (which was not certified by peer review) is the author/funder, who has granted bioRxiv a license to display the preprint in perpetuity. It is made available under aCC-BY 4.0 International license.

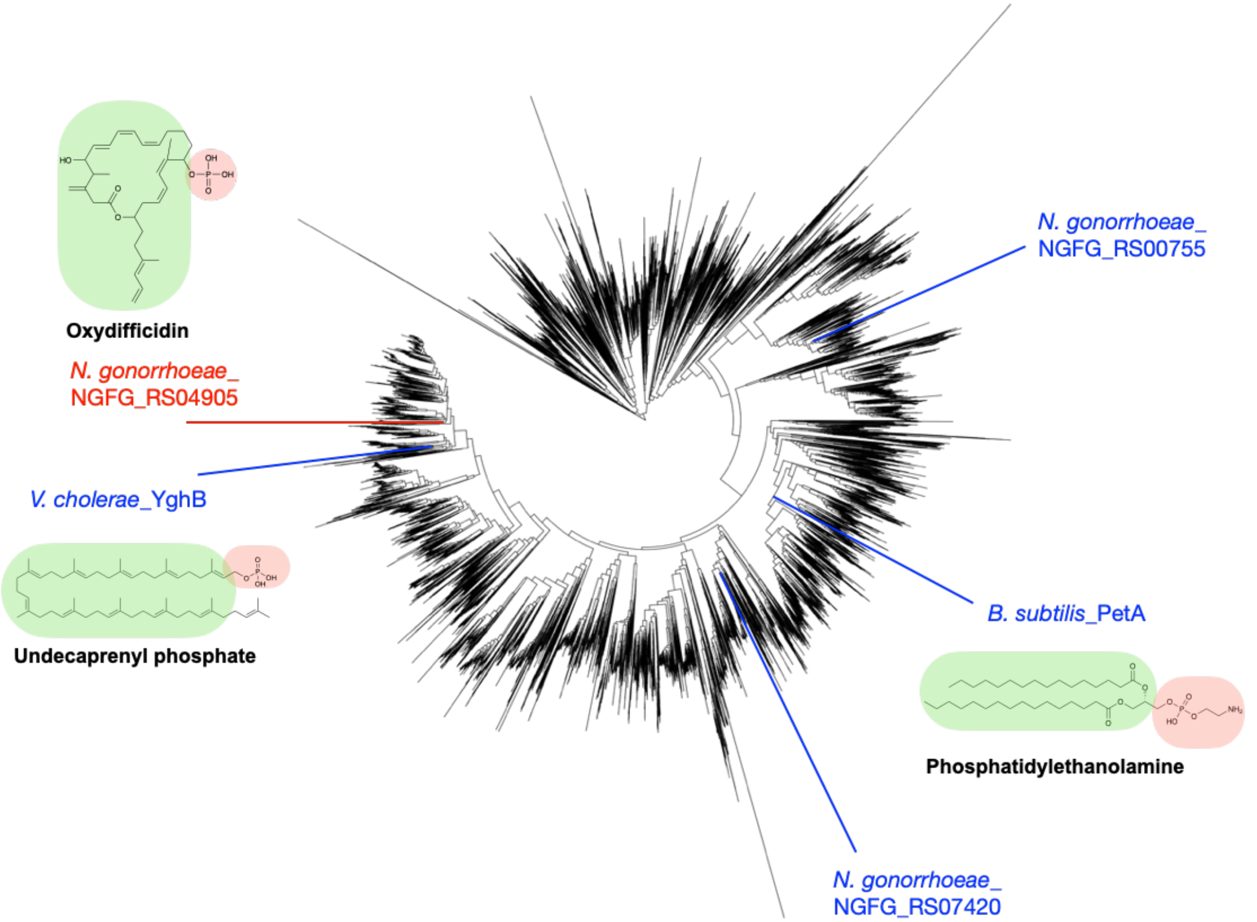

Fig. S2. Phylogenetic tree of 15,825 bacterial DedA family proteins. The tree was built by MUSCLE v5 and FastTree and visualized using iTOL. *N. gonorrhoeae* NGFG_RS04905 highlighted in red represents the DedA gene associated with the activity of oxydifficidin. *N. gonorrhoeae* NGFG_RS07420 and *N. gonorrhoeae* NGFG_RS00755 represents 2 other DedA family proteins in *N. gonorrhoeae*.

bioRxiv preprint doi: https://doi.org/10.1101/2024.05.27.596031; this version posted May 28, 2024. The copyright holder for this preprint (which was not certified by peer review) is the author/funder, who has granted bioRxiv a license to display the preprint in perpetuity. It is made available under aCC-BY 4.0 International license.

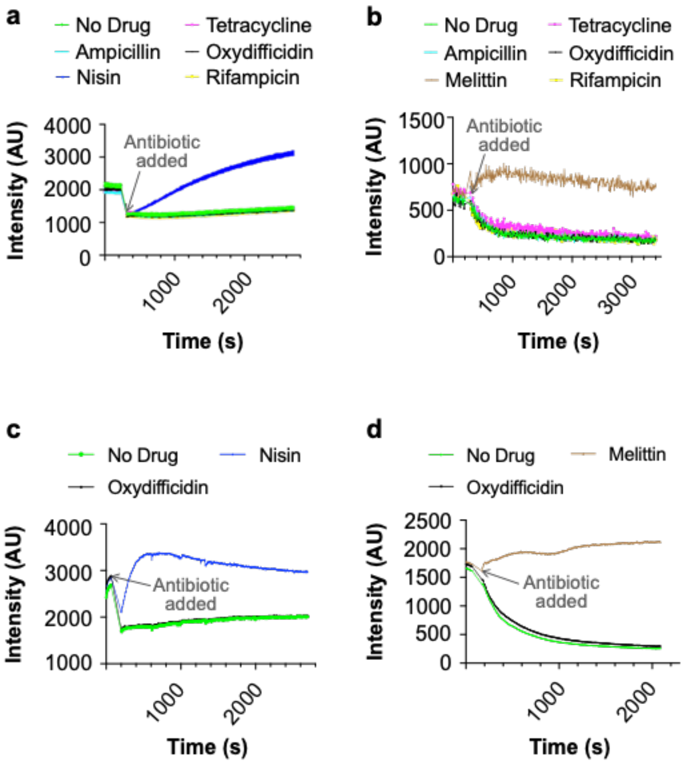

Fig. S3. Oxydifficidin does not lyse or depolarize the membrane of *N. gonorrhoeae*. a. Lysis assay using SYTOX green dye and 8x the MIC of each antibiotic. **b.** Depolarization assay using $DiSC_3(5)$ dye and 8x the MIC of each antibiotic. **c.** Lysis assay using SYTOX green dye with 100x the MIC of oxydifficidin and 32x the MIC of nisin. **d.** Depolarization assay using $DiSC_3(5)$ dye with 100x the MIC of oxydifficidin and the 32x the MIC of melittin. (n = 3 for all assays)

bioRxiv preprint doi: https://doi.org/10.1101/2024.05.27.596031; this version posted May 28, 2024. The copyright holder for this preprint (which was not certified by peer review) is the author/funder, who has granted bioRxiv a license to display the preprint in perpetuity. It is made available under aCC-BY 4.0 International license.

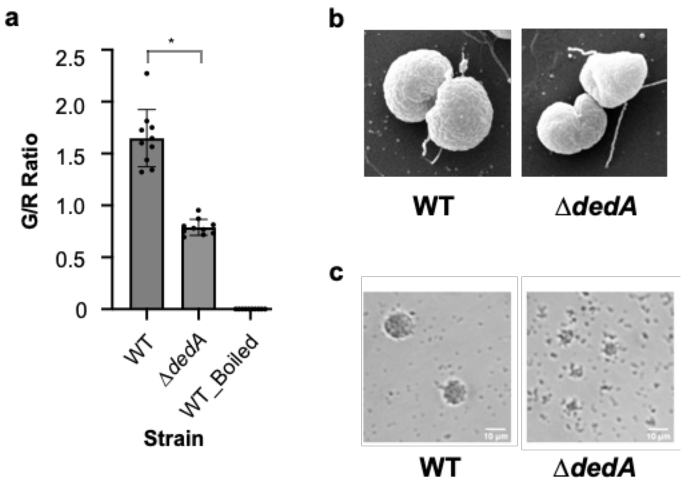

Fig. S4. Mutations in *dedA* affect cell morphology and pili functionality of *N. gonorrhoeae*. a. Membrane integrity assay of *N. gonorrhoeae* WT and *dedA* deletion mutant (Δ*dedA*) cells using SYTO 9 and propidium iodide. Cell integrity was assessed using the ratio of green-stained cell count to red-stained cell count. *: $p < 0.05$ **b.** Scanning electron microscope pictures of *N. gonorrhoeae* WT and Δ*dedA* cells. **c.** Micro-colony formation assay of *N. gonorrhoeae* WT and Δ*dedA* cells.

bioRxiv preprint doi: https://doi.org/10.1101/2024.05.27.596031; this version posted May 28, 2024. The copyright holder for this preprint (which was not certified by peer review) is the author/funder, who has granted bioRxiv a license to display the preprint in perpetuity. It is made available under aCC-BY 4.0 International license.

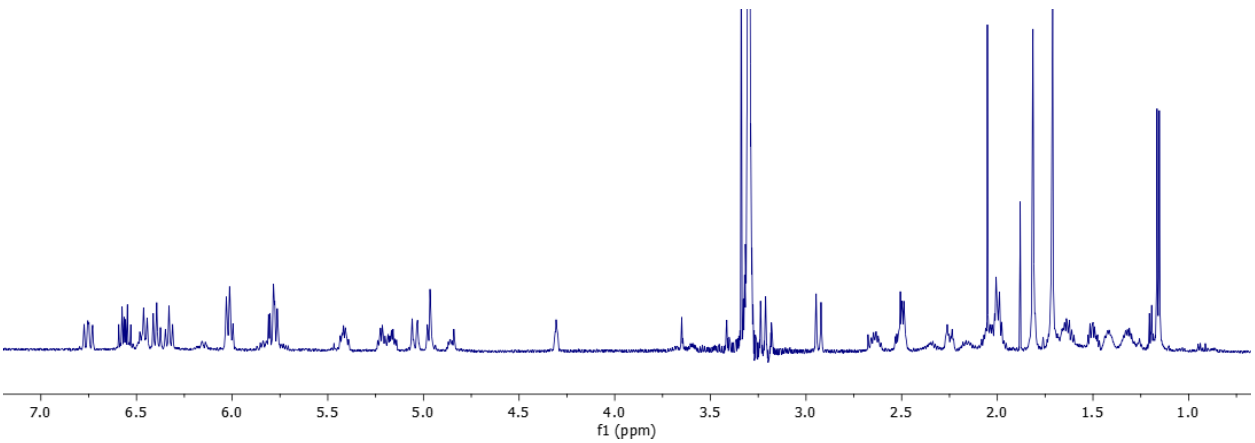

Fig. S5. ^{1}H-NMR spectrum of oxydifficidin (800 MHz, 298 K, CD_3OD - D_2O (1:1)).

bioRxiv preprint doi: https://doi.org/10.1101/2024.05.27.596031; this version posted May 28, 2024. The copyright holder for this preprint (which was not certified by peer review) is the author/funder, who has granted bioRxiv a license to display the preprint in perpetuity. It is made available under aCC-BY 4.0 International license.

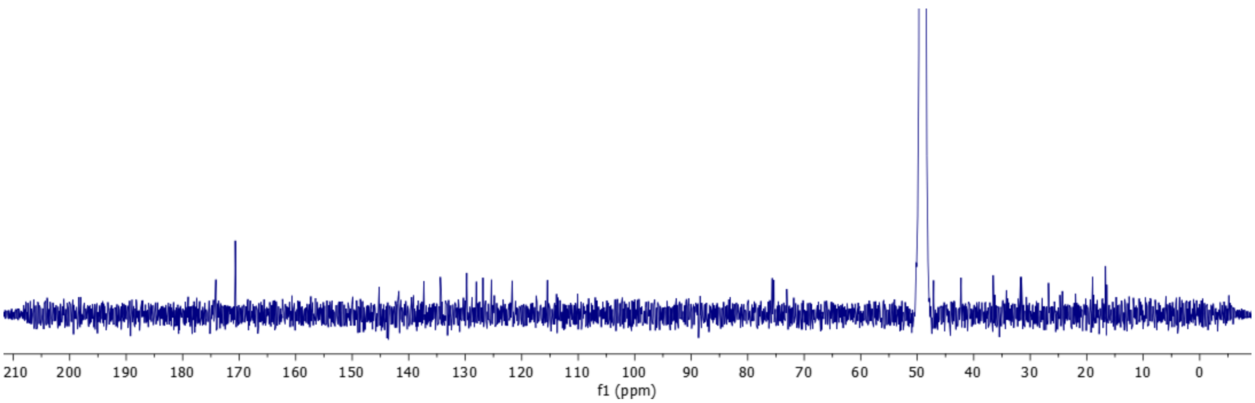

Fig. S6. ^{13}C-NMR spectrum of oxydifficidin (800 MHz, 298 K, CD_3OD - D_2O (1:1)).

bioRxiv preprint doi: https://doi.org/10.1101/2024.05.27.596031; this version posted May 28, 2024. The copyright holder for this preprint (which was not certified by peer review) is the author/funder, who has granted bioRxiv a license to display the preprint in perpetuity. It is made available under aCC-BY 4.0 International license.

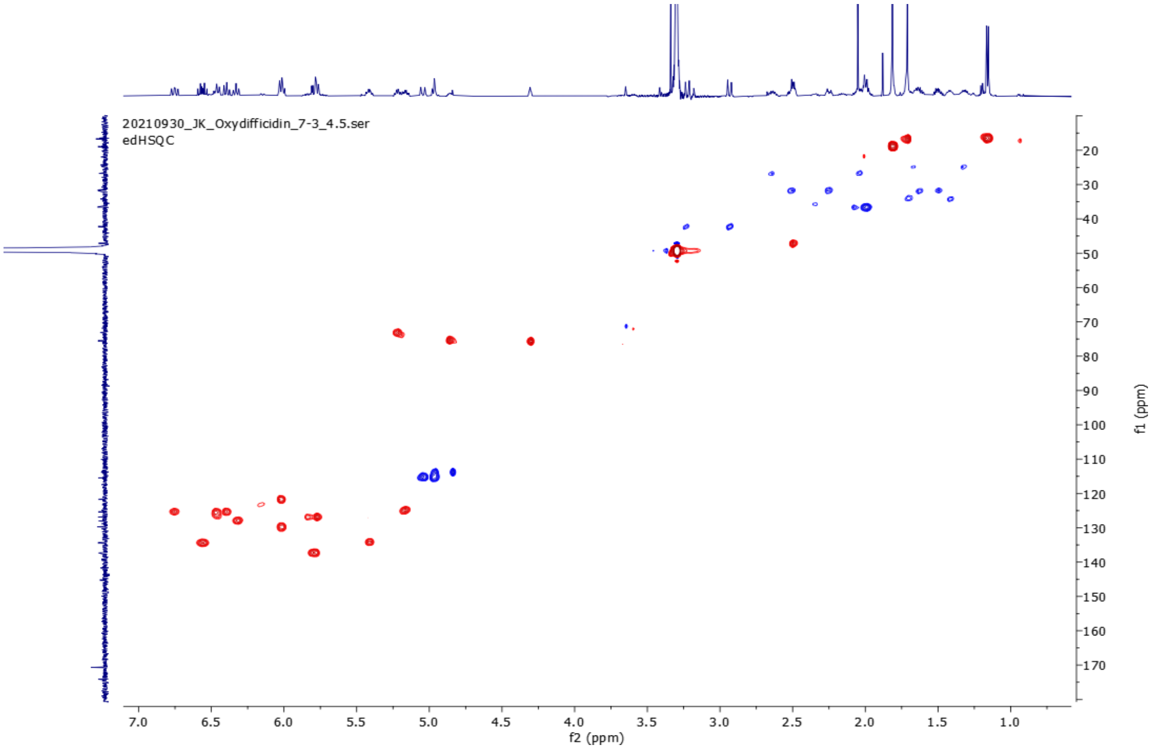

Fig. S7. edHSQC spectrum (800 MHz, 298 K, CD_3OD - D_2O (1:1)) of oxydifficidin.

bioRxiv preprint doi: https://doi.org/10.1101/2024.05.27.596031; this version posted May 28, 2024. The copyright holder for this preprint (which was not certified by peer review) is the author/funder, who has granted bioRxiv a license to display the preprint in perpetuity. It is made available under aCC-BY 4.0 International license.

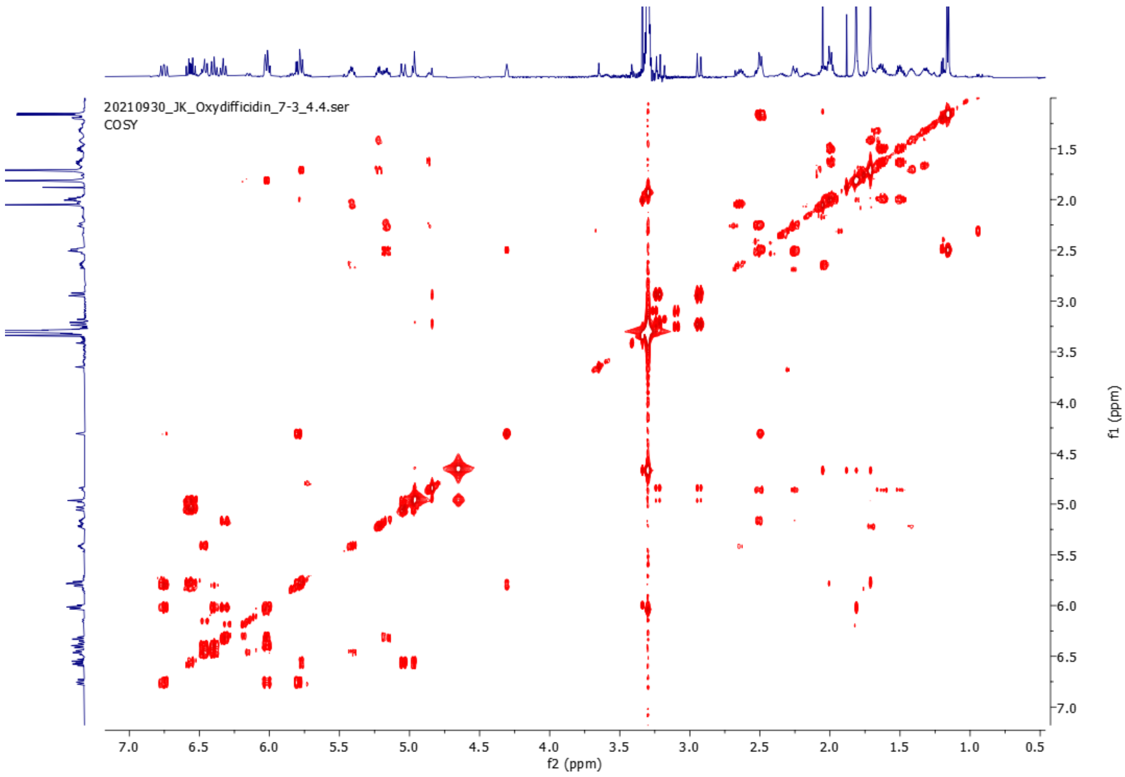

Fig. S8. COSY spectrum (800 MHz, 298 K, CD_3OD - D_2O (1:1)) of oxydifficidin.

bioRxiv preprint doi: https://doi.org/10.1101/2024.05.27.596031; this version posted May 28, 2024. The copyright holder for this preprint (which was not certified by peer review) is the author/funder, who has granted bioRxiv a license to display the preprint in perpetuity. It is made available under aCC-BY 4.0 International license.

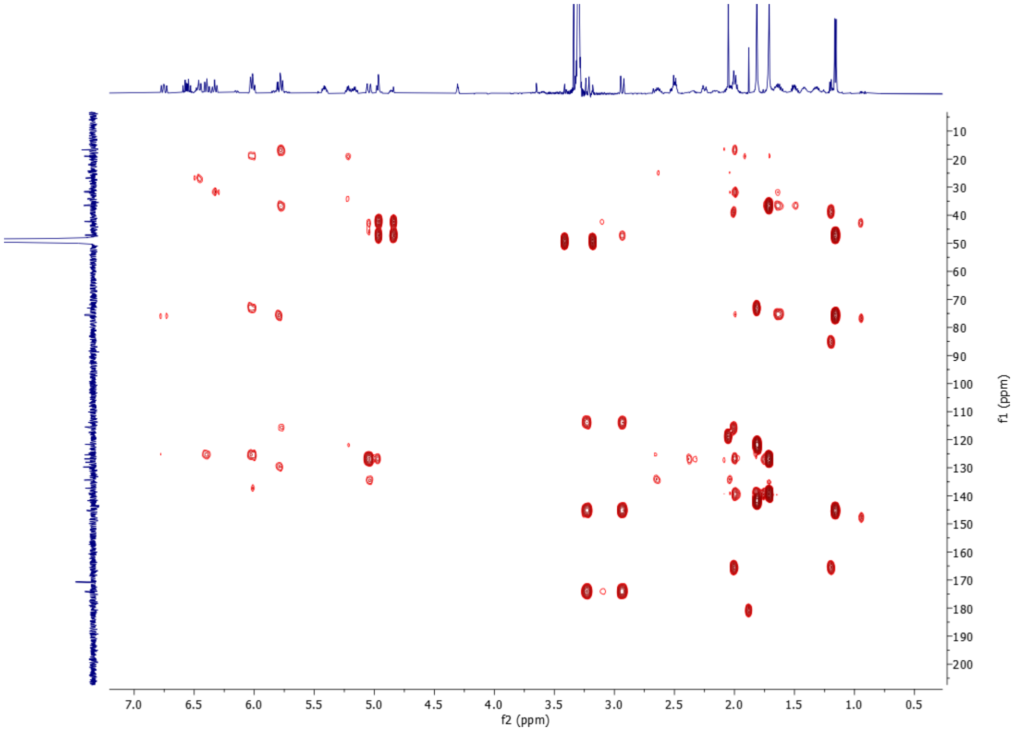

Fig. S9. ^{1}H-^{13}C HMBC spectrum (800 MHz, 298 K, CD_3OD - D_2O (1:1)) of oxydifficidin.

bioRxiv preprint doi: https://doi.org/10.1101/2024.05.27.596031; this version posted May 28, 2024. The copyright holder for this preprint (which was not certified by peer review) is the author/funder, who has granted bioRxiv a license to display the preprint in perpetuity. It is made available under aCC-BY 4.0 International license.

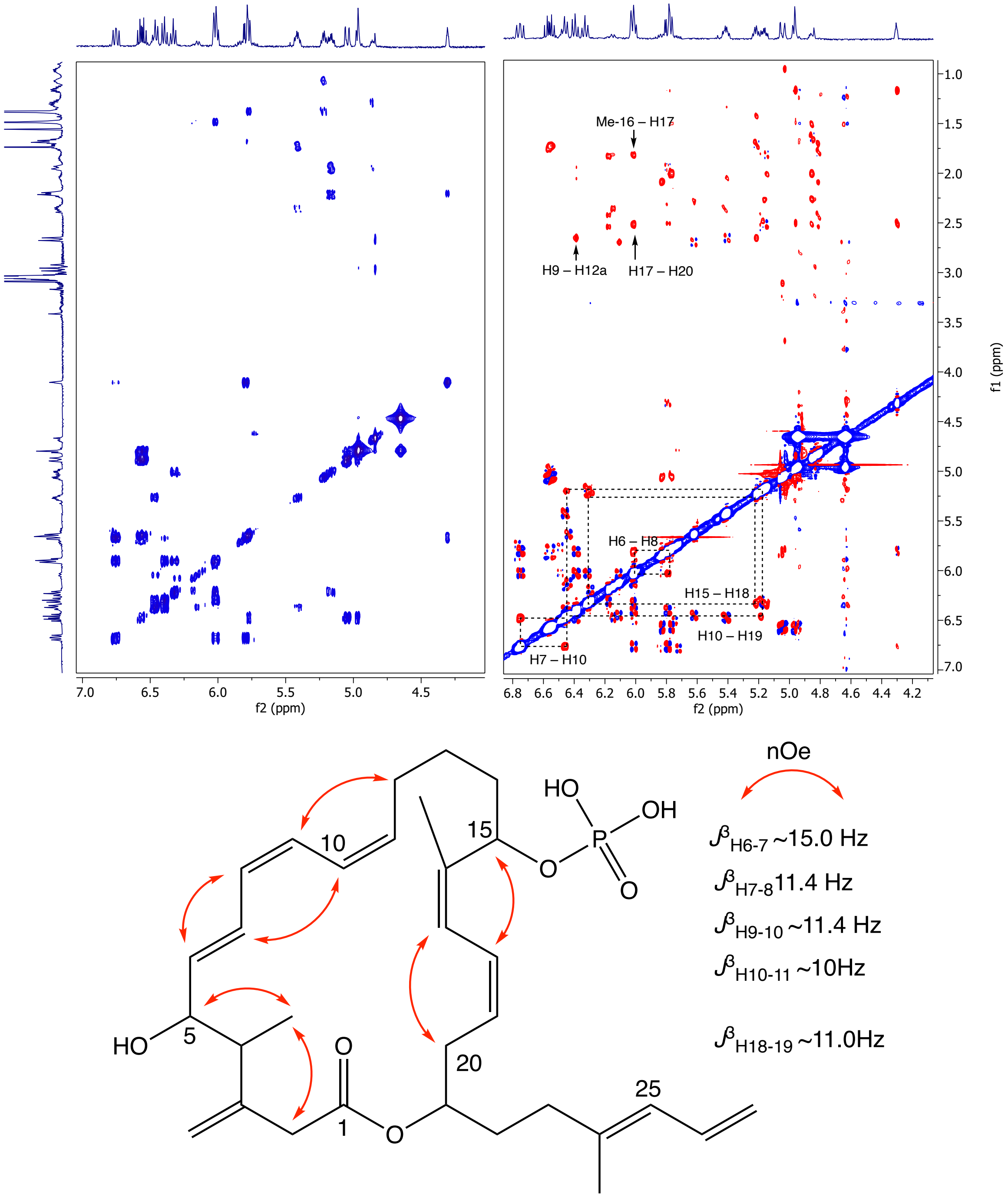

Fig. S10. Partial COSY (left) and ROESY (right) comparison and key ROESY correlations of oxydifficidin (800 MHz, 298 K, CD_3OD - D_2O (1:1)).

bioRxiv preprint doi: https://doi.org/10.1101/2024.05.27.596031; this version posted May 28, 2024. The copyright holder for this preprint (which was not certified by peer review) is the author/funder, who has granted bioRxiv a license to display the preprint in perpetuity. It is made available under aCC-BY 4.0 International license.

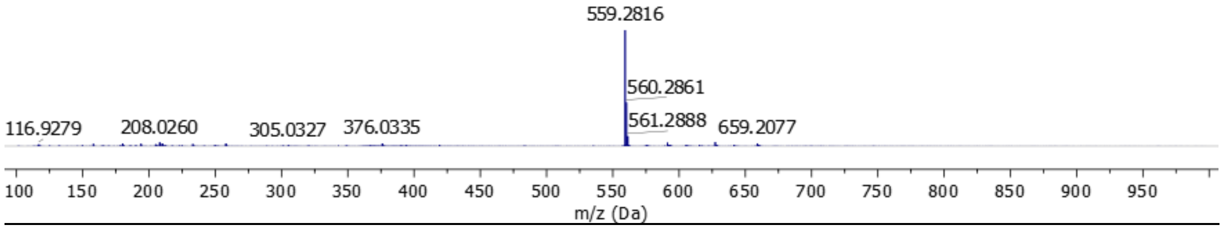

	Ion	Formula	*m/z* calc.	*m/z* obs.	RDB	Δ ppm
1	$[M\text{-}H]^-$	$C_{31}H_{44}O_7P^-$	559.2819	559.2816	11.0	-0.64

Fig. S11a. Full HRMS and annotation of oxydifficidin $[M–H]^–$ parental ion.

bioRxiv preprint doi: https://doi.org/10.1101/2024.05.27.596031; this version posted May 28, 2024. The copyright holder for this preprint (which was not certified by peer review) is the author/funder, who has granted bioRxiv a license to display the preprint in perpetuity. It is made available under aCC-BY 4.0 International license.

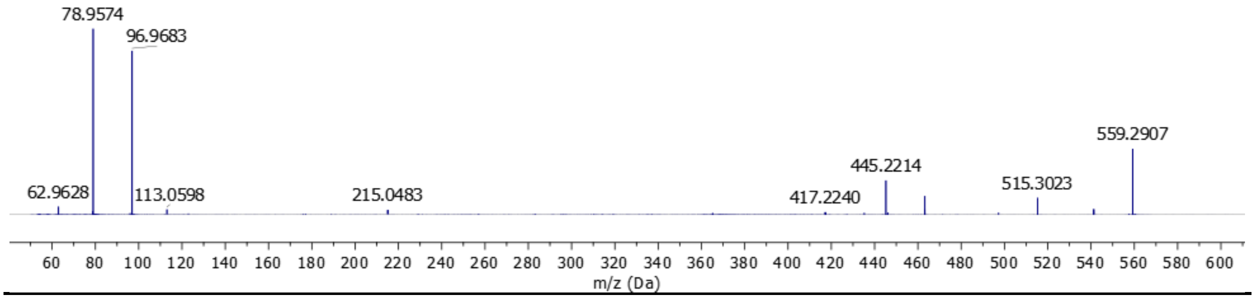

	Ion	Formula	*m/z* calc.	*m/z* obs.	RDB	Δ ppm
	[M-H]$^-$	$C_{31}H_{44}O_7P^-$	559.2830	559.2907	10.5	13.7
a	[X-H-H_2O]$^-$	$C_{30}H_{44}O_5P^-$	515.2932	515.3023	9.5	17.6
b	[X-H]$^-$	$C_{25}H_{35}O_6P^-$	463.2255	463.2328	8.5	15.8
c	[X-H-H_2O]$^-$	$C_{25}H_{34}O_5P^-$	445.2149	445.2214	9.5	14.5
d	[X-H]$^-$	$H_2O_4P^-$	96.9696	96.9683	0.5	13.9
e	[X-H]$^-$	O_3P^-	78.9591	78.9574	0.5	-21.5

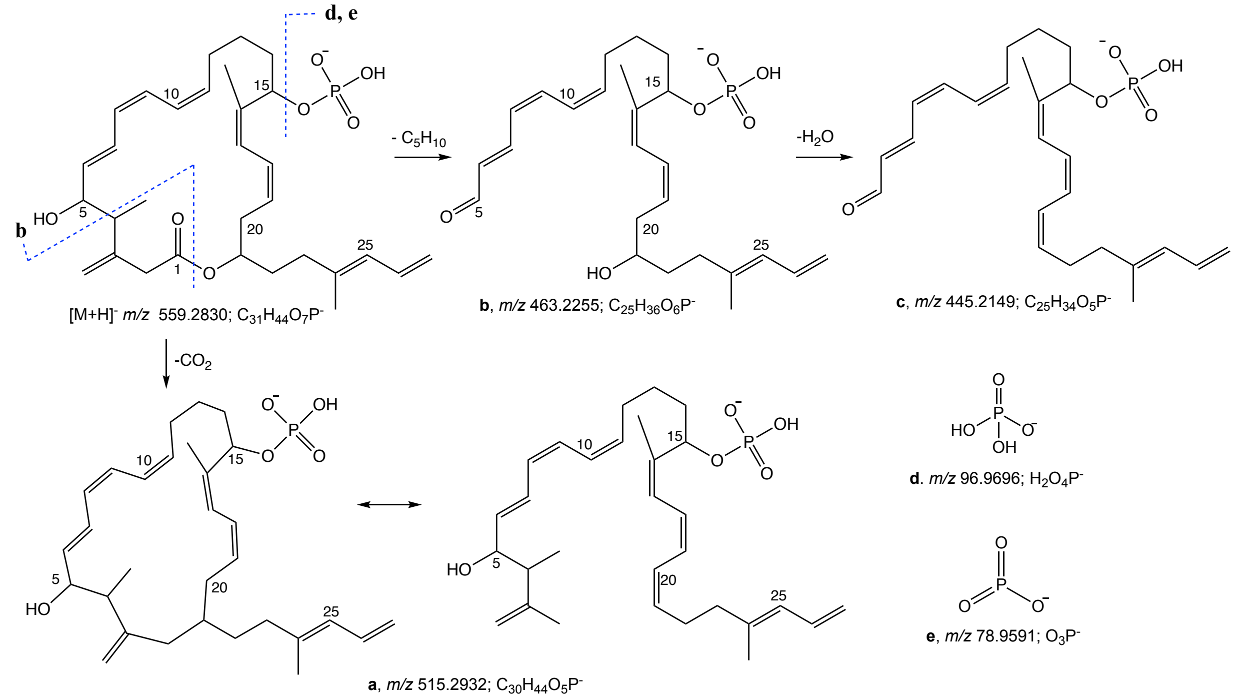

Fig. S11b. -ESI MS/MS spectrum and fragment annotation of oxydifficidin [M–H]$^-$ ion.

bioRxiv preprint doi: https://doi.org/10.1101/2024.05.27.596031; this version posted May 28, 2024. The copyright holder for this preprint (which was not certified by peer review) is the author/funder, who has granted bioRxiv a license to display the preprint in perpetuity. It is made available under aCC-BY 4.0 International license.

Table S1. Activity of oxydifficidin against of Neisseria mutants.

Organism	MIC (µg/ml)*
N. gonorrhoeae MS11	0.25
N. gonorrhoeae MS11 Δ*dedA*	2
N. gonorrhoeae MS11 Δ*dedA rplL*_R76C	16
N. gonorrhoeae MS11 Δ*dedA rplL*_K84E	16
N. cinerea ATCC 14685	2
N. cinerea ATCC 14685 *rplL*_K84E	16
N. subflava NJ 9703	8
N. subflava NJ 9703 *dedA*_I59-D82Del	64
N. subflava NJ 9703 *dedA*_A78-K212Del	64
N. subflava NJ 9703 *dedA*_A160V	64
N. subflava NJ 9703 *dedA*_L53-K212Del	64
N. subflava NJ 9703 *dedA*_Q22*	64
N. subflava NJ 9703 *dedA*_G49S	64
N. subflava NJ 9703 *dedA*_C37*	64

bioRxiv preprint doi: https://doi.org/10.1101/2024.05.27.596031; this version posted May 28, 2024. The copyright holder for this preprint (which was not certified by peer review) is the author/funder, who has granted bioRxiv a license to display the preprint in perpetuity. It is made available under aCC-BY 4.0 International license.

Table S2. ^{1}H and ^{13}C NMR data of oxydifficidin (800 MHz, 298 K, CD_3OD - D_2O (1:1))

Position	Type	^{13}C		^{1}H	multiplicity, *J* (Hz)
1	C	174.1		-	
2	CH_2	42.3	a	3.22	d, 15.5
			b	2.93	d, 15.5
3	C	145.2		-	
3-CH_2	CH_2	113.9	a	4.97	s
			b	4.84	s
4	CH	47.2		2.50	m
4-CH_3	CH_3	16.5		1.16	d, 7.2
5	CH	75.7		4.31	*br* t, 3.5
6	CH	137.3		5.80	dd, 5.5; 15.0
7	CH	125.4		6.75	dd, 11.6; 14.9
8	CH	129.3		6.02	m
9	CH	125.4		6.39	t, 11.4
10	CH	125.4		6.47	t, 11.4
11	CH	134.2		5.41	dt, 5.9; 10.0; 10.0
12	CH_2	26.7	a	2.64	m
			b	2.04	m
13	CH_2	24.8	a	1.67	m
			b	1.32	m
14	CH_2	34.2	a	1.71	br
			b	1.42	m
15	CH	73.1		5.22	dt, 6.3; 8.2; 8.2
16	C	141.8		-	-
16-CH_3	CH_3	18.9		1.81	s
17	CH	121.7		6.02	m
18	CH	128.0		6.33	t, 11.5
19	CH	124.8		5.16	dt, 5.3; 11.0; 11.0
20	CH_2	31.5	a	2.51	m
			b	2.25	dt, 4.0; 4.0; 14.5
21	CH	75.4		4.86	m
22	CH_2	31.7	a	1.63	m
			b	1.50	m
23	CH_2	36.6		2.00	m
24	C	139.2		-	-
24-CH_3	CH_3	16.7		1.72	s
25	CH	126.9		5.77	d, 8.2
26	CH	134.5		6.56	dt, 10.3; 10.3; 16.7
27	CH_2	115.5	a	5.05	dd, 1.7; 16.7
			b	4.97	dd. 1.7, 10.3

^{13}C NMR chemical shifts were obtained by the interpretation of ^{13}C, HSQC and HMBC experiments.

bioRxiv preprint doi: https://doi.org/10.1101/2024.05.27.596031; this version posted May 28, 2024. The copyright holder for this preprint (which was not certified by peer review) is the author/funder, who has granted bioRxiv a license to display the preprint in perpetuity. It is made available under aCC-BY 4.0 International license.

Table S3. Primer Sequences used in this study.

Amplicon	Primer	Sequence (5'-3')
dedA 5' overhang	F	CCCTTTCTGCCTGTACTTCGACTCAAG
	R	CATAAAGTGTCAAGCCCTCGAGGGGTTTTCCAAAACACAATGTCGAGG
dedA 3' overhang	F	GCCGTCTGAAGTTTAAACATCGATTTGTTGGAAATTGACATTATGAATATATTATCCG
	R	CTTGGTAATCGCGCAACAGATCTTCAAGC
trpB-lga 5' overhang	F	AACGCCATCGGTTTGTTCTATC
	R	TAAAGTGTCAAGCCCTCGAGGAGTCAAGCTTCGGACGGCATTTT
trpB-lga 3' overhang	F	GCCGTCTGAAGTTTAAACATCGTTCAGACGGCATTTTATTTTGC
	R	CTTGAGAAGCCGGTTACAAACG
*rplL*_R76C	F	GTGCAACTGGGAAACAATCACA
	R	GTCTTTTTAGGTTACCGCGCTG
kan^R cassette	F	CTCGAGGGCTTGACACTTTATG
	R	ATCGATGTTTAAACTTCAGACGGC
